# Aberrant DNA repair as a potential contributor for the clonal evolution in subsets of anaplastic thyroid carcinomas arising through dedifferentiation: implications for future therapeutic algorithms?

**DOI:** 10.20517/cdr.2020.66

**Published:** 2020-11-03

**Authors:** Carl Christofer Juhlin

**Affiliations:** ^1^Department of Oncology-Pathology, Karolinska Institutet, Stockholm SE-17176, Sweden.; ^2^Department of Pathology and Cytology, Karolinska University Hospital, Stockholm SE-17176, Sweden.

**Keywords:** DNA repair, mismatch repair, P53, thyroid carcinoma, dedifferentiation, anaplastic thyroid carcinoma, clone, treatment

## Abstract

Well-differentiated thyroid carcinoma (WDTC, including papillary thyroid carcinoma and follicular thyroid carcinoma) are fairly slow-growing tumors with an overall low mortality due to the efficacy of combinatory surgery and postoperative radioiodine therapy. Subsets of WDTCs may dedifferentiate into poorly differentiated thyroid carcinoma (PDTC) and anaplastic thyroid carcinoma (ATC), of which especially the latter has an exceptionally poor patient outcome. The underlying genetics responsible for this tumor progression is only partly understood, and is complicated by the fact that subgroups of ATCs are thought to arise *de novo* without a demonstrable, pre-existing WDTC. Even so, recent advances using next generation sequencing (NGS) techniques have identified a genetic link between WDTCs and ATCs, suggesting a step-wise accumulation of mutations driving the loss of differentiation for most cases. In this Commentary, recent findings from an NGS study on synchronous FTC, PDTC, and ATC tumor components from the same patient are highlighted. By using whole-genome data, clonality analyses identified a chief ancestral clone carrying mutations in *TP53*-associated signaling networks regulating genes involved in DNA repair, with sub-clones in each tumor component that were identified also in the less differentiated, neighboring tumor. Moreover, mutational signatures suggested a general mismatch repair (MMR) deficiency along with microsatellite instability. These findings support the chained progression model of dedifferentiation in thyroid cancer, and pinpoint a central role for defective DNA repair. Since effective treatment modalities for ATCs are urgently needed, studies regarding therapeutic agents specifically targeting defective MMR in dedifferentiated thyroid carcinoma could be pursued.

## Introduction

### Anaplastic thyroid carcinoma: a clinical background

Although rare, anaplastic thyroid carcinoma (ATC) is a highly lethal form of thyroid cancer^[1,2]^. Patients are usually in the older span and often present with dramatic symptoms such as a rapidly growing neck mass, dyspnea, and/or hoarseness^[3]^. The diagnosis is usually pinpointed after cytological examinations of fine needle biopsy material, in which highly pleomorphic and undifferentiated tumor cell aggregates are demonstrable^[3]^. The tumor is often metastasized at original presentation, and the current treatment modalities in such cases are mostly palliative in nature, with debulking surgery and combinations of radio- and chemotherapy (cisplatin or doxorubicin), as well as tyrosine kinase inhibitors constituting the most common approaches in the clinical setting^[1,2,4-8]^. Less than 5% of patients with distant metastases survive more than five years; however, the chance of cure is higher if the patient only exhibits localized disease without regional or distant involvement^[7]^. Notably, long-term survival of ATC patients with disseminated disease have also been described, of which some have been treated with tyrosine kinase inhibitors, while rare cases with spontaneous remission also have been reported^[9,10]^.

### Clonal evolution of ATCs

**Figure 1:**
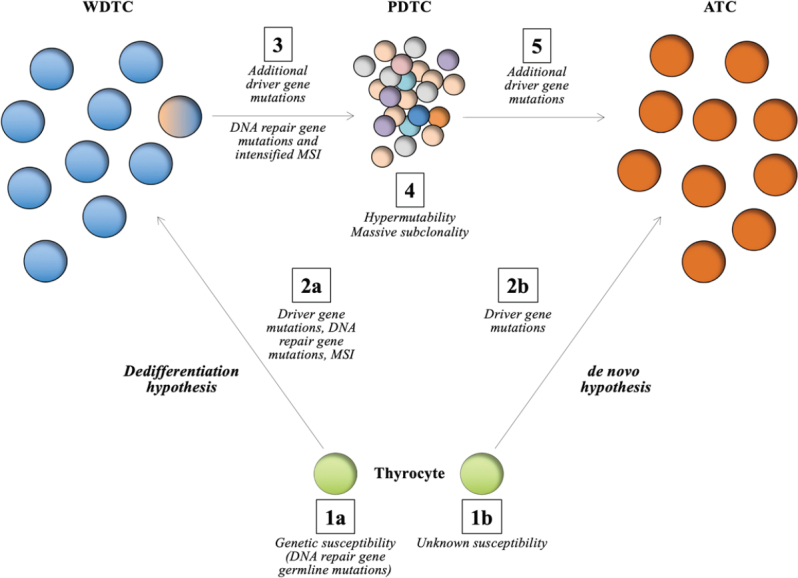
Schematic representation of the genetic mechanisms observed in the evolution of DNA repair defective anaplastic thyroid carcinomas (ATCs) as outlined by Paulsson *et al*.^[26]^. The two main hypotheses regarding formation of ATCs are shown, with most focus on the “dedifferentiation hypothesis”. In thyroid tumorigenesis, the normal thyrocyte is normally afflicted by a set of somatic gene mutations by random chance, possibly also influenced by exogenic factors and underlying rare constitutional variants in susceptibility genes, of which some are associated to DNA repair mechanisms (in this case *MUTYH*) (step 1a-2a). At this point, somatic mutations in DNA repair genes (e.g., *MSH2*) and microsatellite instability (MSI) is evident (step 2a). This leads to the formation of a well-differentiated thyroid carcinoma (WDTC), in this case an follicular thyroid carcinoma. Additional somatic mutations in driver genes and DNA repair genes along with increased MSI in a sub-clone (step 3) most likely influence the formation of a PDTC, which exhibits hypermutability and massive formation of additional tumor sub-clones (step 4), of which subsets of these could transform into an ATC following additional somatic mutations (step 5). The *de novo* hypothesis is outlined in step 1b-2b. Apart from driver gene mutations, little is known regarding the genetics propelling the formation of an ATC directly from a normal thyrocyte, without preceding tumor formations

ATCs have traditionally been thought to develop along two separate paths: (1) *de novo*, without evidence of a pre-existing, well-differentiated thyroid carcinoma [WDTC, including papillary thyroid carcinoma; PTC and follicular thyroid carcinoma (FTC)]; or (2) as dedifferentiated forms of pre-existing WDTCs through an inter-stage poorly differentiated thyroid carcinoma (PDTC) [Figure 1]. The *de novo* theory builds on the fact that patients with ATCs do not always display clinical or histological evidence of an associated WDTC, but as always in medicine - “absence of evidence is not evidence of absence”. As ATCs are highly proliferative lesions with an infiltrative growth pattern and associated inflammation, destruction of nearby tissues could in theory mask the presence of a WDTC in certain cases - making the *de novo* concept less persuasive. Moreover, differentiated forms of thyroid cancers are histologically present in the majority of ATC cases reported in larger studies from multi-center institutions, suggesting a linkage between these tumor forms^[11]^. Arguing in favor of the *de novo* hypothesis, previous cytogenetic analyses have pinpointed wide-spread aneuploidy among ATCs, while concurrently observed WDTCs were largely diploid^[12]^. Although this study vaguely suggests that the tumors might have evolved from different mother clones, a tumoral evolution from WDTC to ATC via progressive genomic instability could not be excluded either.

The advent of high-throughput next-generation sequencing (NGS) techniques such as whole-exome and whole-genome sequencing (WES/WGS) has revolutionized molecular biology, and also increased our understanding of thyroid cancer dedifferentiation tremendously. By recent NGS studies, the mutational landscapes of PDTCs and ATCs have been partly deciphered, and the results indicate that a large proportion of cases exhibit common driver mutations normally seen in WDTCs^[13,14]^. For example, two the most common recurrent mutations seen in PDTCs and ATCs are the V600E *BRAF* mutation as well as hotspot *RAS* mutations, which are mutually exclusive in ATCs (*BRAF* or *RAS*). Those mutations are also frequently observed in PTCs (*BRAF* and *RAS*) and FTCs (*RAS*). The occurrence of similar driver mutations in WDTCs and dedifferentiated forms of thyroid cancer could argue in favor for the chained progression theory, but does not fully exclude the *de novo* hypothesis either. As additional genetic events are seen in PDTCs and ATCs compared to WDTCs, some of these could influence the tumor progression in synergy, not least *TP53* and *TERT* - two of the most commonly mutated genes in ATC^[11,13-16]^. However, data from paired WES studies of WDTCs and ATCs are now accumulating, and the majority of cases seem to exhibit a common and short phylogenetic tree trunk with identical mutational profiles adjoined by disparate mutations as a consequence of the tumors separating early in clonal evolution^[17,18]^. These data strongly imply that ATCs, whenever a synchronous WDTC is visualized, do in fact arise by dedifferentiation from a pre-existing WDTC, highly important information from preventive and therapeutic angles.

### Defective DNA repair in ATCs: lessons from the past

As PDTCs and ATCs in general exhibit an overall higher mutational burden than WDTCs, it is not surprising that researchers are turning their attention to defects in DNA repair systems to explain these differences. Indeed, previous reports have highlighted the potential role of aberrant DNA repair mechanisms in thyroid cancer - not least through the known exogenic coupling between ionizing radiation, oxidative stress, and the development of thyroid neoplasia^[19]^. In fact, patients with WDTC exhibit an overrepresentation of rare variants in genes responsible for DNA repair, including mismatch repair (MMR) genes such as *MSH6*, nucleotide excision repair genes (e.g., *XPC, ERCC5, CCNH*) as well as genes involved in homologous recombination repair (HRR) (e.g., *XRCC3*, *ATM*, *BRCA1*, *CHEK2*)^[20-22]^. Moreover, MMR gene dysregulation and microsatellite instability (MSI) have been found in subsets of WDTCs, adding fuel to the hypothesis that aberrant DNA repair is an important component in thyroid tumorigenesis^[23,24]^.

Through NGS studies in ATC, somatic mutations in DNA repair genes has been reported^[13,14]^. MSI is not uncommon, and subsets of ATCs seem to exhibit a hypermutator phenotype with an immense increase in somatic mutations - most often in tumors with defects in MMR genes, such as *MLH1*, *MLH3*, *MSH5*, and *MSH6*^[13]^. Moreover, these MMR deficient, hypermutated ATCs might display a slightly better overall prognosis than MMR competent ATCs, possibly reflecting a prognostic significance of this genetic feature in clinical practice^[25]^. Overall, ATCs are generally genetically unstable compared to their WDTC counterparts with a massive overrepresentation of gross genetic aberrations, but a specific coupling between ATCs and defective HRR has not yet been established^[12,13]^. As of this, the reason for the increase in gross structural abnormalities observed in ATCs compared to WDTCs is not fully established.

## Whole-genome sequencing as a tool to pinpoint clonal evolution and DNA repair deficiency in thyroid cancer dedifferentiation

### Aim of the study

In order to deepen the understanding of the phylogenetic relations between WDTCs and ATCs as well as to investigate the role of DNA repair in this process, Paulsson *et al*.^[26]^ recently employed WGS to analyze synchronous manifestations of primary FTC, PDTC, and ATC, as well as regional lymph node metastases of the PDTC and ATC components from a single patient. The main aim was to obtain high-resolution phylogenetic data through the interrogation of both mutations as well as copy number alterations on a genome-wide scale, as previous studies focused solely on WES, allowing for a less comprehensive copy number coverage. Moreover, as the tumors analyzed were diagnosed synchronously in the same thyroid lobe from the same patient and operation, this gave the authors a unique opportunity to study the genomic progression of each tumor component without the risk of sampling bias or unrelated patient comparisons. Herein, the overall results from this study as well as associated interpretations are highlighted from both tumor progression and DNA repair perspectives.

### The progression of thyroid cancer as a chained clonal event

In order to obtain material for a comparative analysis between the different thyroid tumor types derived from the single patient, Paulsson *et al*.^[26]^ successfully extracted DNA from normal thyroid tissue as well as neighboring FTC, PDTC, and ATC components, in addition to disseminated PDTC and ATC lymph node metastases. This strategy required the material to be scraped from sections derived from formalin-fixated paraffin-embedded tissues rather than fresh-frozen ones, as the primary FTC, PDTC, and ATC components were bordering each other and only properly visualized using light microscopy.

Following successful whole-genome sequencing and bioinformatics analyses, Paulsson *et al*.^[26]^ identified a number of coding mutational events in cancer-associated genes from each tumor component, which were identified as somatic after correcting for variants also present in constitutional tissue. Some of these mutations were carried along from the FTC all the way to the metastatic ATC, potentially suggesting they provided the tumor components with a selective advantage (i.e., *CALR*, *MSH2*, and *RB1*). Additional tumor-related genes were mutated at the level of the PDTC component, also identified in the neighboring ATC and both metastatic lesions (i.e., *APC*, *DROSHA*, *TP53*, *TERT*, *KMT2A*, and *ASXL1*), implying that one or several of these contributed to the dedifferentiation process. Furthermore, mutations in *TSC1*, *TSC2*, *JAK1*, and *DAXX* appeared at the level of ATC, making it likely that some of these genetic events influenced the development of this undifferentiated tumor type - although only the *TSC1* and *DAXX* mutations were carried along to the metastatic ATC component.

From a clonality perspective, a staggering five-fold increase in mutational burden was noted for the PDTC component compared to the other tumors sequenced, including the ATC. The vast majority of these PDTC mutations were found to be sub-clonal, and they were not carried on to the ATC component [Figure 1]. The authors then employed a computerized clonality analysis; in which impactful mutations in cancer associated genes and whole-genome copy number data were merged to create phylogenetic clusters for each of the different tumor components^[27]^. By doing so, the authors were able to highlight a main, truncal clone that branched off into five major sub-clones. The mutational and copy number backbone in the main clones of the primary ATC and both metastatic samples were identified as small sub-clones present already in the well-differentiated FTC - strongly suggesting that the tumor components were genetically linked. These findings also open up possibilities to identify future markers of potentially lethal sub-clones in cases of WDTC to detect cases at risk for future dedifferentiation, which would be highly warranted from a clinical standpoint. It should be noted however, that non-functional and/or non-coding mutations were not included when assessing clonality, which was deemed impossible given the sheer data volume. The authors were thus not able to detail the clonal compositions using passenger alterations, which could have been useful in separating chained clonal distribution patterns of driver gene mutations with associated co-mutations from the *de novo* driver gene mutations occurring in the downstream tumor components.

### The potential role of aberrant DNA repair in thyroid cancer dedifferentiation

Paulsson *et al*.^[26]^ identified a truncating, heterozygous *MSH2* mutation in the FTC that was carried along to all subsequent tumors analyzed. Notably, the *MSH2* gene locus displayed copy-neutral LOH but retained MSH2 protein expression in the FTC, although the downstream tumor components were negative for MSH2 expression. Thus, the authors speculate that the copy neutral LOH must have preceded the *MSH2* mutation in the FTC, followed by a second genetic event in the PDTC and ATC components leading to abolished MSH2 expression - perhaps by promoter hypermethylation of the wildtype allele. All tumor components exhibited MSI, with the lowest score in the FTC, and analysis of the mutational composition showed a substantial accumulation of C>T transitions compared to A>G transitions and transversions for all tumor components - a known hallmark for deficient MMR. Several significant signatures were correlated to deficient MMR when correlating the genetic data to established COSMIC mutational signatures. By *in silico* gene ontology analyses of impactful coding mutations in the mother clone (from which all major sub-clones in the tumor components emerged), Paulsson *et al*.^[26]^ identified “*TP53* associated transcription of DNA repair genes” as the main mutated signaling pathway. In all, these data suggest that the mutational inactivation of *MSH2* gave rise to an MMR deficient profile for all tumor components.

In addition to the truncating *MSH2* mutation, a missense *ATM* mutation was observed in the adjacent PDTC component. *ATM* encodes a kinase protein that regulates signaling pathways involved in DNA repair and overall genome stability through regulation of HRR, including *TP53* associated signaling networks^[28]^. Interestingly, as the PDTC exhibited a hypermutator phenotype compared to all other tumor components, one cannot exclude a synergic effect of combined *MSH2* and *ATM* mutations driving the hypermutability. Of note, *ATM* mutations have been found recurrently in ATCs^[14]^. Even so, as both *ATM* and *TP53* gene mutations are recurrent events also in HRR competent cancer types, there was not enough evidence to suggest that the ATC sample studied was indeed HRR deficient. Furthermore, when analyzing rare constitutional variants in cancer-associated genes by interrogating the normal thyroid sample, the authors detected a homozygous *MUTYH* variant (rs3219468) with a minor allele frequency of < 0.04% in the general population. Interestingly, *MUTYH* is involved in DNA repair, especially response to oxidative DNA damage^[29]^. Whether this rare variant in any way influenced the development of multiple thyroid tumors in this patient is not known.

### Relevance for future therapeutic interventions

Given the dismal overall prognosis and the limited treatment options for ATC patients, numerous *in vitro* and clinical studies focusing on various adjuvant therapies have been launched in recent years, including studies using immunotherapy (i.e., antibodies targeting PD-L1 and/or PD-1), epigenetic silencing (i.e., histone deacetylate inhibitors), metabolic pathway interference (i.e., stearoyl-CoA desaturase 1 inhibitors), as well as specific gene therapies using an adenovirus vector^[5]^. Immunotherapy seems especially promising given the high frequency of PD-L1 and PD-1 positive ATC cells in clinical samples^[30]^, as well as initial studies suggesting partial response or stable disease in subsets of patients after immunotherapy^[31,32]^. Interestingly, in unrelated tumor types, the expression of PD-L1 and the prognosis of patients receiving immune checkpoint inhibitors have been found to be influenced by the MMR status and somatic mutations in MMR genes^[33-36]^. If the same is true for ATC patients is not currently known, but given the known association between aberrant MMR and the development of this highly lethal tumor type, studies on this topic are highly warranted.

In colorectal cancer, there is an association between MMR-deficient tumors and an observed resistance to various chemotherapeutic drugs used, not least 5-fluorouracil^[37]^. For this reason, numerous studies have been conducted in order to identify additional therapeutic options for cancers with an MMR deficiency. In one study, the authors selectively knocked-down the function of DNA polymerases *POLB* and *POLG* in cell lines deficient in *MSH2* and *MHL1* respectively, causing oxidative DNA lesions and lethal double strand breaks^[38]^. Moreover, cytarabine, a cytosine nucleoside analogue, has been proposed as an alternative oxidative stress-generating compound with selectivity towards MMR deficient cell lines^[39]^. Interestingly, the folic acid antagonist methotrexate might work in a similar way, as studies have shown an increased lethality for *MSH2* mutated tumors via the induction of oxidative stress and extensive DNA damage^[40]^. To build on this, studies depicting partial responses in ATC patients administered oral methotrexate in combinations with other cytotoxic drugs have been reported, but the MMR status of these tumors was never characterized^[41,42]^. Finally, one should not overlook the potential of gene therapy, for example the usage of out-of-frame “suicide genes”. When introduced in MMR deficient cells, the gene is randomly reverted back to its in-frame version due to the hypermutability, leading to apoptosis^[43]^.

## Discussion

Recent advances in NGS techniques have facilitated the genomic interrogation of thyroid tumors, allowing for a detailed description of the mutational and chromosomal landscapes. This in turn has increased our understanding of common driver events in thyroid tumorigenesis as well as highlighted markers of diagnostic, prognostic, and therapeutic value in the clinical setting.

In the study outlined above, the authors amassed evidence for the progressive model, as thyroid tumors were clonally linked, further supporting the theory that thyroid cancer dedifferentiation in this case was a chained genetic event rather than synchronous manifestations of tumors arising separately by random chance. Moreover, it seems tempting to speculate that a proportion of ATCs develop as a consequence of aberrant DNA repair mechanisms due to somatic inactivation of MMR genes in pre-existing WDTCs, in turn leading to hypermutability and the generation of multiple tumor sub-clones, of which some might give rise to an undifferentiated phenotype [Figure 1]. In this aspect, the progression of ATC could be looked at as a “genetic tombola”, in which hypermutability in preceding tumor forms (in our case exemplified by a PDTC) will generate a massive amount of sub-clones, of which single ones will acquire additional tumor-propagating mutations and/or gross chromosomal events, thus gaining a selective advantage and emerging as highly lethal ATCs. However, it should be stated that the majority of ATCs do not exhibit hypermutability and defective MMR, and therefore additional genetic and/or epigenetic events are most probably responsible for the dedifferentiation in these cases.

ATCs are feared tumors in the clinical setting, not least due to the limited response to conventional treatment options^[2,6]^. The advent of molecular testing has helped identify subset of tumors with potentially targetable mutations, not least mutations in *BRAF*, *mTOR*, and *ERBB2*. Even though partial responses have been noted, exceedingly few patients survive once the tumor spreads to distant sites^[13,14]^. The notion that a proportion of ATCs exhibit MMR deficiency should therefore not be overlooked, as MMR deficient tumors could mandate specific therapeutic considerations, and as the hypermutability in turn might give rise to additional targetable mutations^[44]^. Especially the promising value of immune checkpoint inhibitors in subsets of ATCs should be evaluated against the MMR status of these tumors, as the hypermutability in MMR deficient ATCs in theory would generate a massive overrepresentation of “non-self” tumoral antigens, in turn priming the patient’s immune response. However, as most ATCs lack hypermutability and MMR deficiency, the identification of additional molecular mechanisms suitable for targeted therapies is still highly warranted for the majority of cases.

To conclude, ATC patient outcome is exceedingly poor when distant spread is evident. While conventional treatment options are largely ineffective, targeted molecular therapy has shown promising results in terms of prolonged survival even after disease dissemination. The knowledge that subsets of ATCs probably arise as a clonal evolution from DNA repair malfunctioning precursor tumors could potentially be of importance when considering adjuvant treatment options for this ill-fated patient category, not least through direct or indirect targeting of a defective MMR system.

## Declarations

### Copyright

© The Author(s) 2020.
